# 

**Published:** 1876-05

**Authors:** 


### Table of Contents.

### ORIGINAL COMMUNICATIONS.

Does Alcohol Afford Nutrition to the Animal Tissues? *By J. P. Stevens, M.D., of Lee county* .... 65
A Case of Opium Poisoning. *By A. R. Alley, M.D., Atlanta, Ga.* ... 73
Heart-Clots and Embolism, and Esmarch's Bandage in Amputation. *By John P. Wall, M.D., of Tampa, Fla.* .... 74
A Case of Compound Urinary Fistula. *By V. H. Taliaferro, M.D., Atlanta* .... 83
Ergot in Parturition—Enlarged Spleen. *By A. J. Shaffer, M.D., Gainesville* .... 88

### ATLANTA ACADEMY OF MEDICINE.

Chloroform in Labor—Use of Ergot—Cystic Tumor—Hemorrhage following the use of the Ecraseur—Muscular Tissue in Blood-Vessels—Cystitis—Milk-Leg .... 91

### OUR EXCHANGES.

Lister's Treatment of Wounds by the Antiseptic Method .... 98
Bellevue Hospital—Notes of Practice .... 101
The Pathology of Sunstroke .... 105
Mode of Applying Iodine to the Interior of Cysts .... 106
Hospital Reports—New York Woman's Hospital .... 108
Use of Nelaton's Catheter .... 109
Sequel to the Case of Habitual Constipation .... 110
Chloral in Pityriasis .... 111
Systemic Infection from Purulent Vaginal Discharges .... 112
Where to make Hypodermic Injections ....
Removal of Foreign Bodies from the Ear .... 113
The Turpentine Treatment of Enteric Fever .... 114
Spontaneous Rabies .... 115
Irritability of the Bladder Treated by Dilatation of the Urethra ....
Pills for Obstinate Neuralgia .... 116
Gleanings ....

### BIBLIOGRAPHICAL.

A Manual of General Pathology .... 118
Medical Thermometry and Human Temperature ....

### EDITORIAL.

Georgia Medical Association .... 119
In Memoriam .... 124
Dr. Ralph Walsh's Call Book .... 128

